# Methyl Fluoride Affects Methanogenesis Rather than Community Composition of Methanogenic Archaea in a Rice Field Soil

**DOI:** 10.1371/journal.pone.0053656

**Published:** 2013-01-14

**Authors:** Anne Daebeler, Martina Gansen, Peter Frenzel

**Affiliations:** 1 Department of Biogeochemistry, Max Planck Institute for Terrestrial Microbiology, Marburg, Germany; 2 Department of Microbial Ecology, Netherlands Institute of Ecology (NIOO-KNAW), Wageningen, The Netherlands; University of Waterloo, Canada

## Abstract

The metabolic pathways of methane formation vary with environmental conditions, but whether this can also be linked to changes in the active archaeal community structure remains uncertain. Here, we show that the suppression of aceticlastic methanogenesis by methyl fluoride (CH_3_F) caused surprisingly little differences in community composition of active methanogenic archaea from a rice field soil. By measuring the natural abundances of carbon isotopes we found that the effective dose for a 90% inhibition of aceticlastic methanogenesis in anoxic paddy soil incubations was <0.75% CH_3_F (v/v). The construction of clone libraries as well as t-RFLP analysis revealed that the active community, as indicated by *mcrA* transcripts (encoding the α subunit of methyl-coenzyme M reductase, a key enzyme for methanogenesis), remained stable over a wide range of CH_3_F concentrations and represented only a subset of the methanogenic community. More precisely, *Methanocellaceae* were of minor importance, but *Methanosarcinaceae* dominated the active population, even when CH_3_F inhibition only allowed for aceticlastic methanogenesis. In addition, we detected *mcrA* gene fragments of a so far unrecognised phylogenetic cluster. Transcription of this phylotype at methyl fluoride concentrations suppressing aceticlastic methanogenesis suggests that the respective organisms perform hydrogenotrophic methanogenesis. Hence, the application of CH_3_F combined with transcript analysis is not only a useful tool to measure and assign *in situ* acetate usage, but also to explore substrate usage by as yet uncultivated methanogens.

## Introduction

Methanogenesis is the dominating terminal process in anoxic freshwater habitats like sediments and flooded soils. In rice fields, most labile organic carbon is derived from plant material, and carbohydrates are the primary source for anaerobes resulting eventually in acetate and H_2_ + CO_2_ as most important methanogenic precursors [1]. The theoretical ratio of acetate : H_2_ + CO_2_ usage equals 2 : 1 [2]. However, depending on the exact oxidation state of labile organic carbon, but also on competing microbial processes, this ratio may vary. Hence, the fraction of methane produced *via* acetate is an important variable in understanding what controls mineralization in anoxic environments.

The amount of acetate-derived methanogenesis can be assessed with CH_3_F (methyl fluoride, fluoromethane), a specific inhibitor for aceticlastic methanogenesis. When applied for the first time in microbial ecology, CH_3_F was assumed to be a specific inhibitor for methane oxidation and ammonium oxidation [3], [4]. While providing direct access to processes, inhibitor experiments may be misleading, if specificity is confined to certain conditions [5]. Indeed, CH_3_F turned out to be an efficient inhibitor of methane and ammonium monooxygenases. However, it soon became evident that it may also inhibit methanogenesis [6], [7]. In anoxic incubations treated with CH_3_F, approximately as much acetate accumulates as methane is lacking compared to untreated controls. Selectivity of CH_3_F for suppression of aceticlastic methanogenesis was further validated in pure culture studies demonstrating that 1% v/v inhibited growth of and methanogenesis by pure cultures of aceticlastic *Methanosaeta* and *Methanosarcina*. Other microbes, homoacetogenic, sulfate reducing and fermentative bacteria, and a methanogenic mixed culture based on hydrogen syntrophy, were not inhibited [7]. In *Methanosarcina barkeri*, which is able to use acetate and H_2_ + CO_2_ simultaneously, only acetate utilization was suppressed, when both acetate and hydrogen were supplied [7]. However, pure cultures are not necessarily representative for yet uncultured populations, and many operational taxonomic units (OTUs) have been designated to a phylogenetic clade and named from environmental sequence information alone. Hence, some populations may show a behavior different from that found in pure cultures.

Another approach to determine methanogenic pathways uses isotopic signatures; for review see [8]. In short, methanogenesis from H_2_ + CO_2_ discriminates stronger against isotopically heavier carbon than does aceticlastic methanogenesis [8], [9]. This difference can be used to calculate the contribution of these two methanogenic pathways, provided the respective isotopic fractionation factors are known [8], [10], [11]. Indeed, combining the application of CH_3_F with the analysis of isotopic signatures revealed the expected patterns [12].

The methanogenic community in rice fields mainly consists of versatile *Methanosarcinaceae* and strictly acetotrophic *Methanosaetaceae*, as well as of hydrogenotrophic *Methanomicrobiales*, *Methanobacteriales*, and *Methanocellales*; the latter were formerly known as rice cluster I [1], [8], [12]–[14]. Rice paddy soil is found to be compartmented into two habitats: rhizosphere and bulk soil. Methanogenic communities on rice roots are dominated by *Methanocellales*, with hydrogenotrophic methanogenesis contributing 60–80% to total methane production [15]–[17]. The influence of rice cultivars was found to be minor [18]. In bulk soil however, methane is mainly derived from acetate (50–83%), and *Methanosarcinaceae* are the prevailing methanogens [19], [20]. The community structure of methanogens remains rather stable even under dry-wet cycles [21]. In summary, cell numbers fluctuate with management [21], but methanogenic communities in paddy fields of different geographical origin are highly related [22].

Here, we re-visit the inhibition of aceticlastic methanogenesis in a paddy soil asking not only how specifically CH_3_F inhibits aceticlastic methanogenesis, but also for the response of different methanogenic archaea to this inhibitor. We studied the dose-response relationship of methanogenesis as a function of CH_3_F concentration by combining process measurements with isotopic data and molecular analyses targeting the *mcrA* gene (encoding the subunit A of methyl coenzyme M reductase, a protein characteristic and essential for methanogenesis [23]). Since quite often only a minor fraction of a methanogenic community is metabolically active [16], [25], [26], we aimed at both the *mcrA* gene (community) and the respective mRNA (active community), as *mcrA* transcripts have been shown to be directly connected to energy metabolism and methanogenesis [24].

## Materials and Methods

One kg bulk soil was sampled in spring 2008 from a rice field in the delta region of River Yangtze (Zhejiang Province, China) representing one of the major rice growing areas of the world. The particular field had been used for wetland rice production for about 2000 years [27]–[29]. Ten grams air-dried soil were mixed with ten milliliters oxygen-free distilled water in 26-ml pressure tubes. Tubes were capped with butyl rubber stoppers and flushed with N_2_ for ten minutes. Different amounts of CH_3_F corresponding to initial concentrations of 0.2, 0.3, 0.4, 0.6, 0.79, 0.99, 1.19, 1.57, 1.96, 2.72 and 3.85% were injected by syringe in two tubes each. Another three tubes did not receive CH_3_F serving as control, and three were sampled immediately as primary soil material. Water, tubes, and stoppers had been sterilized. The tubes were incubated for14 days in the dark at 25°C. Methane, carbon dioxide and methyl fluoride in the headspace were measured repeatedly after sampling with a 0.25-ml pressure-lok syringe (Valco Instruments, USA) on a GC-FID (SRI-8610, SRI Instruments, USA). Only endpoint measurements are shown here. Quantification of lactate, formate, acetate, propionate, ethanol and butyrate were performed by analyzing filtered (ReZist, 0.2 µm PTFE, Schleicher and Schuell, Germany) pore water samples after 14 days of incubation by HPLC (SRI Instruments, USA).

Methane produced from carbon dioxide (m_CO2_) was measured under inhibition of aceticlastic methanogenesis (≥0.75% CH_3_F, see below), while methane produced from acetate (m_acetate_) was calculated from the balance to total methane produced in controls without inhibitor: m_acetate_ = m_total_−m_CO2_.

Carbon isotopic signatures in methane and acetate were measured as described elsewhere [30]. ^13^C signatures are given in δ-notation referring to the respective standard material, Vienna Pee Dee Belemnite (VPDB) [8].

Total nucleic acids were extracted as described elsewhere [31]. For tRFLP analysis, *mcrA* gene fragments were obtained with primers ME1/ME2 [32], where the forward primer was labeled with FAM. PCR conditions were: initial denaturing at 94°C for 5 minutes, 35 cycles of 30 s at 94°C, 45 s at 55°C, 1.5 min at 72°C, and a final extension at 72°C for 5 min. Amplicons were digested with SAU96I and analyzed on a capillary sequencer (3130 Genetic Analyzer, Applied Biosystems). For reverse transcriptase PCR (RT-PCR), 5 µl sample were treated with DNA-free DNase (Qiagen) followed by exonuclease treatment (mRNA-ONLY Prokaryotic mRNA Isolation Kit, Epicentre Technologies) and cleaning (RNAeasy Mini Kit, Qiagen) according to manufacturers' instructions. Reverse transcription and amplification was performed in one step combining reverse transcription (Reverse Transcription System, Promega, Germany) with 30 PCR cycles at conditions as described above, but without a FAM-label on primer ME1.

**Figure 1:**
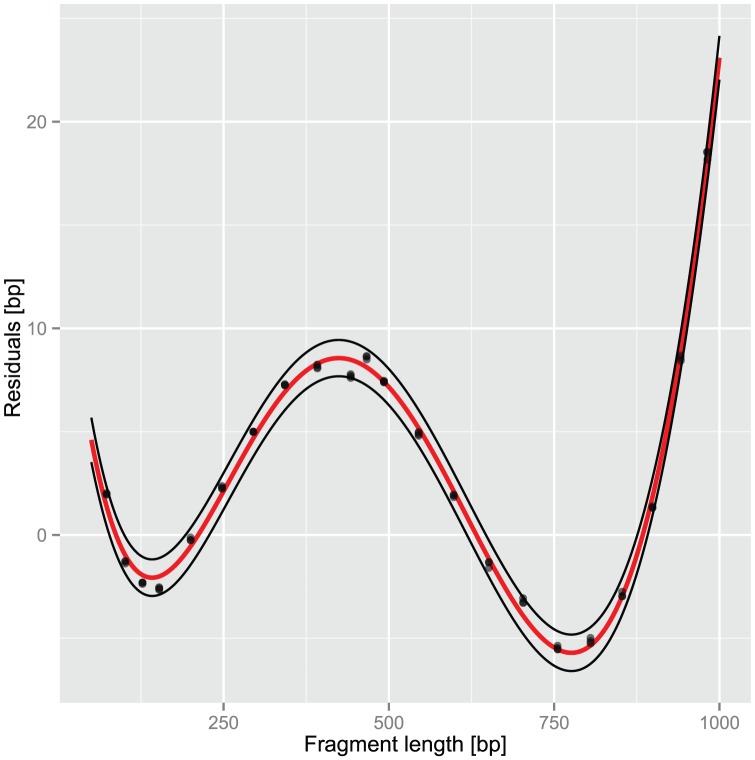
Residuals, the difference between real and estimated size, of a FAM-labeled size standard used as ‘sample’ in t-RFLP analysis. Data from three replicate runs are shown. Fit: fifth order polynomial, red line; 95% prediction intervals: black lines.

In tRFLP analysis measured fragment size may deviate from real (*in silico*) size. Different factors have been claimed to be responsible for size shifts [33], [34], but a detailed residual analysis was lacking so far. Residuals, the difference between real and estimated size, were calculated by running a FAM-labeled size standard as ‘sample’ against a ROX-labeled size standard. Both standards were purchased from Eurogentec (Germany). The ‘fragment’ size of the FAM-labeled standard was calculated with the built-in software using a third order polynomial as calibration function. Even if the calibration curve gave nearly perfect fit, residuals showed a considerable non-linearity being best described by a fifth order polynomial (Figure 1; intercept = 16.67359, a = −0.3238648, b = 1.831838e-3, c = −3.81772e-06, d = 3.17735e-09, e = −8.61187e-13). This polynomial was used to correct measured TRF size making it comparable to *in-silico* fragment size.

Gene libraries for archaeal *mcrA* sequences were constructed using cDNA from the control samples and from samples incubated under 3.85% methyl fluoride, as well as DNA from the primary soil material. (RT)-PCR products were ligated into pGEM-T vector plasmids (Promega, Germany) and transformed into *Escherichia coli* competent cells JM109 (Promega, Germany) according to the manufactures' instructions. The sequences were assembled with SeqManII (DNASTAR) and compared with sequences available in the GenBank database using the BLAST network service to determine the approximate phylogenetic affiliations. Alignment and phylogenetic analysis of the *mcrA* sequences from 69 DNA- and 91 mRNA-derived clones was done with ARB [35]. OTUs were defined by the average neighbor algorithm at 5% amino acid sequence divergence level; representative sequences for these OTUs were determined using mothur ver. 1.19.3 [36]. Sequence data have been submitted to GenBank under accession numbers JQ283291-JQ283438.

Statistical analysis was done in R ver. 3.12.2 [37]. Dose-response models were fitted using package drc, ver. 2.2-1 [38]. Constrained correspondence analysis (CCA) and non-metric multidimensional scaling (NMDS) were done with package vegan ver. 2.1-0 [39], and a multivariate regression tree (MRT) was fitted with package mvpart ver. 1.4-0 [40]. Graphics were produced with package ggplot2 [41].

## Results and Discussion

### Metabolites and isotopic signatures

**Figure 2:**
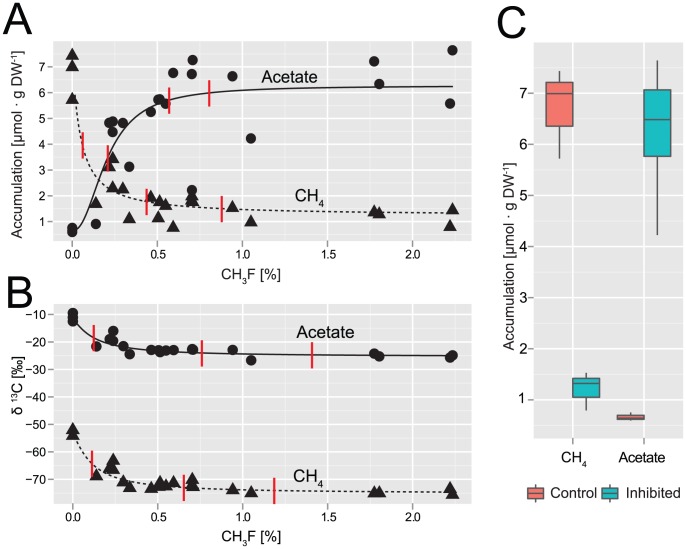
Accumulation of acetate and methane (A), and the respective δ^13^C signatures in ‰ VPDB (B) depending on initial concentrations of methyl fluoride; δ^13^C_acetate_ is the combined signature for both C-atoms. Data are endpoint measurements and not corrected for initial concentrations. The fitted dose-response curves follow a log-logistic model with the parameters ED_50_ (effective dose for 50% inhibition), upper limit, and slope, while the lower limit was fixed to the respective averages for 0% CH_3_F. ED_50_, ED_90_, and ED_95_ are marked by red lines. (**C**) Box-plot summarizing accumulation of methane and acetate in control (n = 3) and in samples with CH_3_F≥0.75%, n = 6) after 14 days of anoxic incubation.

With increasing CH_3_F concentration, acetate accumulated while methane accumulation was reduced accordingly (Figure 2A) resulting in a highly significant negative correlation (r = 0.7, P = 0.0002). No other fermentation products, in particular not formate, propionate, butyrate, or ethanol, did accumulate (data not shown). Along with the reduction of methanogenesis, both the δ ^13^C values of methane and acetate decreased (Figure 2B). The shift in δ ^13^C-CH_4_ by about −20‰ VPDB between control (0% CH_3_F) and incubations receiving ≥0.75% CH_3_F is in accordance with a shift from mixed substrate usage to H_2_ + CO_2_ dependent methanogenesis [17], [42]. Correspondingly, the relatively heavy carbon isotopic signature of −10‰ in acetate from control incubations implies that lighter acetate was preferentially consumed, thus enriching the remaining acetate in ^13^C. With increasing CH_3_F concentration, δ^13^C_acetate_ continuously decreased until values stabilized around −23‰, as known for acetate derived from fermentation of organic matter in rice fields [12]. Thereby we can exclude that homoacetogenesis was an important process in the incubations, as otherwise the isotopic signature of acetate should have been substantially lower [43].

All fitted dose-response curves have ED_90_ (effective dose for 90% inhibition) concentrations of <0.75% CH_3_F. The dose-response curves for acetate and methane accumulation even showed ED_99_ concentrations of <1%. The higher ED_99_ for the isotopic signatures may be due to the rather gentle slope of the respective curves (Figure 2B).

If only aceticlastic methanogenesis was inhibited while acetogenesis proceeded, the sums of methane and acetate in control and fully inhibited samples (assumed at ≥0.75% CH_3_F) should be equal. Indeed, no significant difference was found (Figure 2 C; two sample t-test, p = 0.87). On basis of the results of the different dose-response curves we conclude additionally that above 0.75% CH_3_F virtually no acetate was consumed. Furthermore, our data does not indicate an effect on residual, hydrogenotrophic methanogenesis. In a previous experiment, hydrogenotrophic methanogenesis was found unaffected even at 4% CH_3_F [6: in a hypersaline microbial mat from Solar Lake, Sinai]. However, in two incubations at elevated CH_3_F concentrations (2.7 and 2.9%) not included in the dose-response fits, the amount of acetate produced was about 50% higher than the corresponding methane deficit. Methanogenesis and isotopic signatures, on the other hand, were not affected. Similar disproportionate acetate values have been reported before [44] and perhaps, these imbalances are caused by substrate heterogeneities, not by effects on methanogenesis.

Assuming that an initial CH_3_F concentration of 0.75% inhibited aceticlastic methanogenesis, hydrogenotrophic methanogenesis contributed 18.3% to total methane production. The inhibitory concentration is within the range usually applied to rice field [2], [4], [6], [17], [44]–[49] and other wetland soils [50]–[53]. A decade ago, CH_3_F was thought to be a specific inhibitor for methane oxidation in general [3] and has been applied to chamber experiments quantifying methane oxidation from the difference between methane fluxes with and without CH_3_F (Table 1). Considering an ED_50_ of <0.25% CH_3_F for aceticlastic methanogenesis, these experiments may likely have underestimated the amount of methane oxidized due to co-inhibition of aceticlastic methanogenesis.

**Table 1:** Experiments quantifying methane oxidation from the difference between methane fluxes measured with and without CH_3_F.

Reference	Year	Ecosystem	Biome, Ecozone	CH_3_F concentration
[45]	1995	Wetland rice	Temperate	1%
[58]	1997	Wetland	Temperate	1.5%
[47]	1996	Wetland rice	Tropics	1.5, 3%
[59]	1993	Wetland rice, weeds	Subtropics	1.5, 3%
[6]	1996	Wetland rice	Mediterranean	0.7, 1.7, 3%
[60]	2001	Weed (*Myriophyllum*)	Temperate	84–140 µM
[48]	2001	Wetland rice	Subtropical	3%
[61]	1996	Weed (*Sparganium*)	Boreal	3–4%
[62]	1998	Tundra wetland	Subarctic	1%
[63]	2000	Wetland	Boreal	1.5–3%

### The methanogenic community

**Figure 3:**
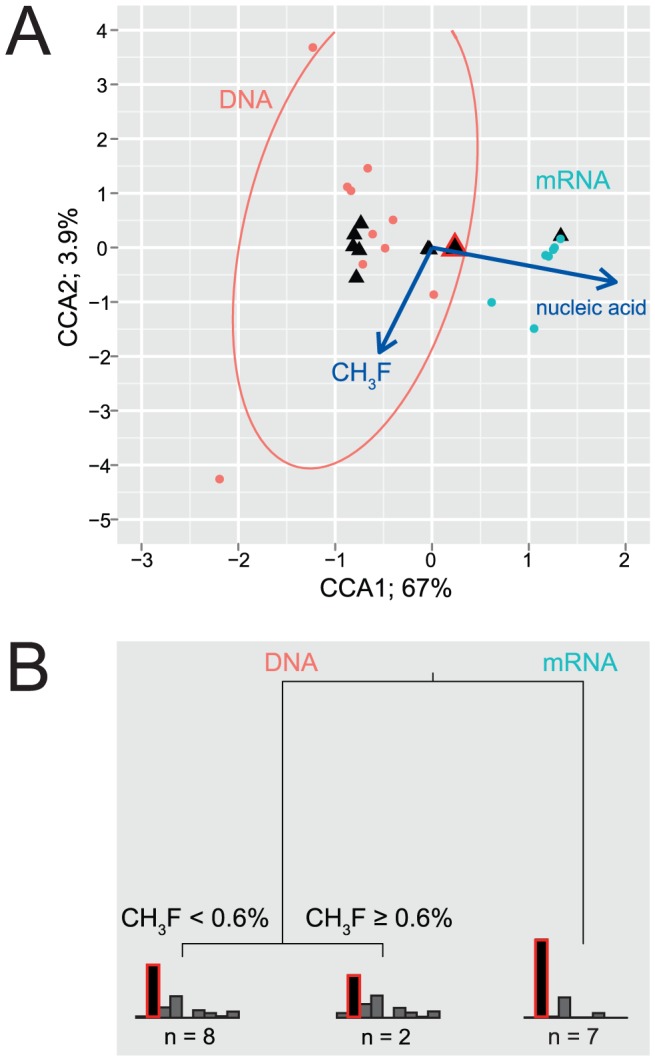
Multivariate analysis of relative abundances of terminal restriction fragments (tRF). (**A**) Biplot of a constrained correspondence analysis (CCA). Two constraints were applied: CH_3_F concentration and the type of nucleic acid, *i.e.* DNA or mRNA. The CCA explains about 71% of overall variation, with CCA1 being the most important axis. The arrows indicate the direction in which constraints correlate with the ordination axes. Confidence ellipses (95%) surround the centers of DNA- and mRNA-derived communities, respectively. Closed circles represent the samples, and black triangles the different tRFs. The triangle surrounded by a red outline corresponds to tRF 133, the numerically dominant fragment. (**B**) Multivariate regression tree (MRT) based on squared Euclidean distances. The vertical spacing of the branches is proportional to the error in the fit; the first split reduces the error by 75%. The tree is pruned, i.e. the least important splits have been removed. Barplots at the leaves show the relative abundance of different tRFs; from left: 126, 133, 503, 648, 652, 663, 683, 743, and 752 bp. As in panel A, tRF 133 is marked by a red outline.

Community composition (DNA-based) and transcripts were analyzed by t-RFLP analysis as well as by cloning of the *mcrA* gene fragments and transcripts. Results of the t-RFLP analysis of the *mcrA* gene (Figure 3) indicated a high relative abundance of versatile *Methanosarcinaceae* (tRF 126, 133, 652, 683) and hydrogenotrophic *Methanobacteriales* (tRF 126, 663, 752). In addition, *Methanocellales* (tRF 133) were found in all incubations. Two tRFs could not be separated further: an *in silico* analysis of *mcrA* sequences from the clone library revealed that tRF 133 occurred in *Methanocellales*, the Fen cluster, and *Methanosarcinaceae*, while tRF 126 comprised both *Methanobacteriales* and *Methanosarcinaceae*. Despite this, t-RFLP patterns showed a distinct separation between total and active community in all analyses applied: CCA (Figure 3A) and MRT igure 3B) demonstrated consistently that a homogenous, active community was found across the whole CH_3_F gradient applied. Furthermore, virtually the same separation was found with non-metric multidimensional scaling (NMDS; stress = 0.02, r^2^_linear_ = 0.99; ordination not shown). As found recently for methanogens [21] and other microbial guilds [54], the active community consisted only of a subset of the total. Most remarkable was here the nearly complete absence of restriction fragments indicative for *Methanobacteriales mcrA* transcripts.

**Figure 4:**
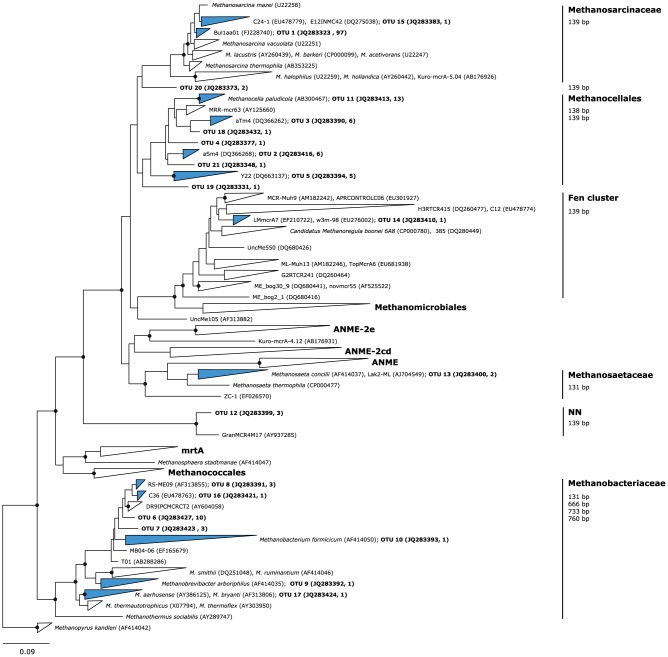
Neighbor-joining tree based on 147 deduced amino acid positions from 949 *mcrA* sequences. Phylogenetic nodes verified by a maximum likelihood tree are marked with closed circles. The outer branches of distinct clusters are collapsed, and those containing OTUs defined in this study are marked in blue. Only representative sequences for the OTUs have been incorporated into the tree and are depicted as ‘OTU name (accession number, number of sequences representing the OTU)’. Environmental clusters were labeled with two reference sequences showing maximum phylogenetic distance within the respective cluster, given as ‘name 1 (accession number 1), name 2 (accession number 2). The corresponding tRFs were calculated *in silico* using the TRiFLe package [64] and are given to the right. Scale bar: 0.09 changes per amino acid position. The outgroup is *Methanopyrus kandleri*.

Cloning and sequencing allowed further differentiation. The DNA-based library constructed from soil sampled at the beginning of the experiment was dominated by sequences affiliated to *Methanocellales*, *Methanosarcinaceae* and *Methanobacteriales*, but also by a few members of the Fen cluster and a so far uncharacterized cluster (Table 2). The latter (OTU 12; Table 2) were found in clones retrieved under CH_3_F suggesting a hydrogenotrophic mode of life. In accordance with our t-RFLP findings, only a minor fraction of this diversity could be retrieved from mRNA resulting in highly significant differences between DNA- and mRNA-based clone libraries (Table 2). Considering mRNA derived sequences as a proxy for group-specific activity, *Methanobacteriales* appeared to not produce methane at all. Similarly, *Methanocellales* seemed to have been much less important for methanogenesis than expected from their high dominance in the DNA-based clone library. With and without repression of aceticlastic methanogenesis, *Methanosarcinaceae* were the most active methanogens suggesting that they used acetate when possible, but shifted to H_2_ + CO_2_, if acetate usage was inhibited. This is in accordance with a previous experiment on *Methanosarcina barkeri* strain MS that was inhibited by CH_3_F when supplied with acetate, but not if grown on H_2_ + CO_2_ [7]. *Methanosarcinaceae* sequences detected here were affiliated to the type strain of *Methanosarcina mazei* (Figure 4) being able to use both these substrates, too [55]. It is intriguing that under CH_3_F inhibition, no *Methanocellales*-related sequences could be retrieved anymore from mRNA, resulting in a small yet still significant difference between the respective libraries (Table 2). While we cannot rule out a direct effect, shifting *Methanosarcinaceae* towards a hydrogenotrophic mode of life might also have changed competition for H_2_ resulting in an indirect effect on *Methanocellales*.

**Table 2:** Abundances of the 22 operational taxonomic units (OUTs) with a maximum intra-group distance of 5% (AA) in the clone library.

OTU	Affiliation	TRF	Start, DNA	Control, mRNA	CH3F 3.85%, mRNA
1	Msarc	139	15	40	42
2	Mcell	139	6	0	0
3	Mcell	138	6	0	0
4	Mcell	139	1	0	0
5	Mcell	139	5	0	0
6	Mbac	760	10	0	0
7	Mbac	131	3	0	0
8	Mbac	760	3	0	0
9	Mbac	666	1	0	0
10	Mbac	666	1	0	0
11	Mcell	138	10	3	0
12	NN	139	1	0	2
13	Msaeta	131	2	0	0
14	Fen	139	1	0	0
15	Msarc	139	1	0	0
16	Mbac	733	1	0	0
17	Mbac	760	1	0	0
18	Mcell	138	1	0	0
19	Mcell	138	0	1	0
20	Msarc-like	139	0	2	0
21	Mcell	139	0	0	1
χ^2^ test,simulated p-values				Control, mRNA	CH3F, mRNA
Start, DNA				0.0001	0.0001
Control, mRNA					0.05

Clones were derived from samples taken before (‘start’, based on DNA) and after (‘control’ and 3.85% CH_3_F, based on transcripts) anoxic incubation for 14 days. OTU number and affiliation to families are given as in Figure 4. Msarc: *Methanosarcinaceae*, Mcell: *Methanocellales*, Mbac: *Methanobacteriales*, Msaeta = *Methanosaetaceae*, Fen = Fen cluster, Msarc-like = uncertain affiliation, but nearest to *Methanosarcinaceae*; NN = unknown cluster. Simulated p-values are from a Monte-Carlo simulation with 9999 replicates.

## Conclusion

While we found CH_3_F to act specifically on aceticlastic methanogenesis, the results obtained from the analysis of *mcrA* transcripts allow for relevant conclusions beyond this technical aspect. Community composition has often been regarded as a controlling factor for the flow of carbon and reductants through microbial communities. However, this experiment has shown how versatile *Methanosarcinaceae* are very well capable of delivering the same end-product under totally different conditions. This supports concepts developed to understand and predict the reaction of microbial communities to environmental changes [56], [57]. Furthermore, this experiment demonstrates how the sensible application of selective inhibitors can help detecting physiological traits of yet uncultivated microbes eventually supporting the design of cultivation strategies. Having found previously the same effect of CH_3_F on methanogenesis in a soil from an Italian rice field [6] more than 10,000 km apart from that in China let us trust that our findings are widely applicable.
